# Supplementary material for: Overexpression of the NEK8 kinase inhibits homologous recombination
Source: bioRxiv. 2025 Feb 8:2025.02.07.637121. Preprint. [Version 1] doi: 10.1101/2025.02.07.637121 (PMC11839122; doi:10.1101/2025.02.07.637121)
Supplement: Supplement 1 [file NIHPP2025.02.07.637121v1-supplement-1.pdf]

bioRxiv preprint doi: https://doi.org/10.1101/2025.02.07.637121; this version posted February 8, 2025. The copyright holder for this preprint (which was not certified by peer review) is the author/funder, who has granted bioRxiv a license to display the preprint in perpetuity. It is made available under aCC-BY 4.0 International license.

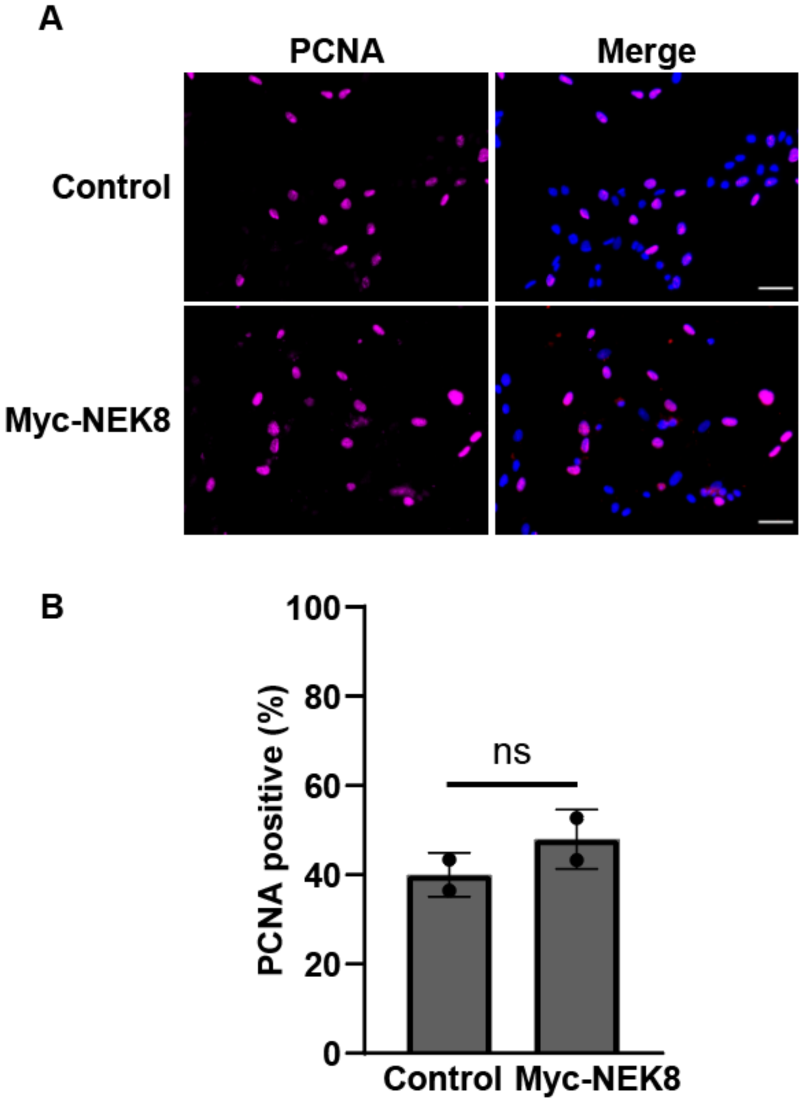

Figure S1. NEK8 overexpression does not alter the percentage cells in S-phase. (A) Representative images of PCNA (magenta) and DAPI (Blue), scale bar = 50μm. **(B)** Bar graph of PCNA positive cells. Dots represent individual data points from independent experiments. N=2. ANOVA; Tukey HSD.

bioRxiv preprint doi: https://doi.org/10.1101/2025.02.07.637121; this version posted February 8, 2025. The copyright holder for this preprint (which was not certified by peer review) is the author/funder, who has granted bioRxiv a license to display the preprint in perpetuity. It is made available under aCC-BY 4.0 International license.

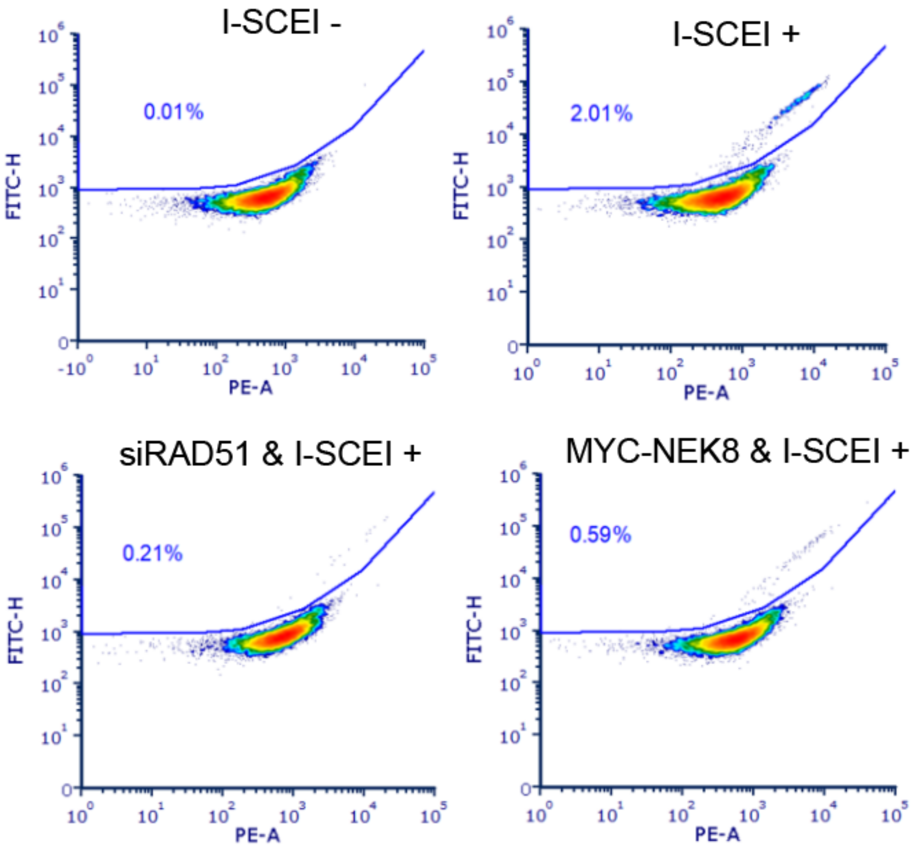

Figure S2. DR-GFP assay to measure HR (HR+). Representative intensity plots depicting HR positive (GFP+) cells in the indicated samples. X-axis is PE, y-axis is GFP. Gates defining GFP+ population is shown in blue. Quantification of assay is in Figure 2B.
